# TECHNOLOGY THAT SUPPORTS EXTENDING DEMENTIA-FRIENDLY COMMUNITY BASED CARE: A SCOPING REVIEW

**DOI:** 10.1093/geroni/igae098.3896

**Published:** 2024-12-31

**Authors:** Nina Buckley, Elizabeth Hewitt, Limei Chen, Sang Choi

**Affiliations:** George Mason University, Fairfax, Virginia, United States; George Mason University, Fairfax, Virginia, United States; George Mason University, Fairfax, Virginia, United States; George Mason University, Fairfax, Virginia, United States

## Abstract

With global aging on the rise, there is increasing interest in technological interventions that support persons living with dementia (PLwD) to age in place. This scoping review identifies and examines technologies specifically designed for dementia care, explores their usage contexts, and assesses their potential for implementation in community settings. A systematic search was conducted across nine electronic databases—APA PsycInfo, AgeLine, MEDLINE, SocINDEX, Global Health, Web of Science, Google Scholar, and CINAHL—following the WHO (2023) framework for the classification of digital health interventions. The review included peer-reviewed articles published between 2014 and 2024, using search terms related to “dementia,” “digital technology,” “quality of life,” and “community.” Articles were screened for eligibility based on predefined criteria, such as being peer-reviewed and written in English. Out of 1,009 articles originally identified, 54 met the inclusion criteria and were included in the review. The findings reveal a range of technologies aimed at keeping PLwD safe and supported at home. However, the majority of studies highlighted a significant reluctance among PLwD to adopt these technologies, often due to a lack of familiarity and comfort with digital tools. This hesitancy, particularly among family caregivers, emerged as a major barrier to the successful implementation of technological interventions in community settings. To enhance the effectiveness of technology in dementia care, there is a need for strategies that address caregiver concerns and improve digital literacy. Future research should focus on developing user-centered interventions that are adaptable to the diverse needs and preferences of both PLwD and their caregivers.

